# Association of general and central obesity, and their changes with risk of knee osteoarthritis: a nationwide population-based cohort study

**DOI:** 10.1038/s41598-023-30727-4

**Published:** 2023-03-07

**Authors:** Dojoon Park, Yong-Moon Park, Seung-Hyun Ko, Kang-Se Hyun, Youn-Ho Choi, Dong-Uk Min, Kyungdo Han, Hae-Seok Koh

**Affiliations:** 1 Department of Orthopedic Surgery, St. Vincent’s Hospital, College of Medicine, The Catholic University of Korea, 93, Jungbu-daero, Paldal-gu, Suwon-si, Seoul, Gyeonggi Republic of Korea; 2 Department of Epidemiology, Fay W. Boozman College of Public Health, University of Arkansas for Medical Sciences, Little Rock, AR USA; 3 Department of Internal Medicine, St. Vincent’s Hospital, College of Medicine, The Catholic University of Korea, Seoul, Republic of Korea; 4 Department of Statistics and Actuarial Science, Soongsil University, Seoul, Republic of Korea

**Keywords:** Diseases, Health care, Risk factors

## Abstract

In this study, we aimed to evaluate the association between general and central obesity, and their changes with risk of knee osteoarthritis (OA) using retrospective cohort data collected from the Korean National Health Insurance Service. We studied 1,139,463 people aged 50 and over who received a health examination in 2009. To evaluate the association between general and/or central obesity and knee OA risk, a Cox proportional hazard models were used. Additionally, we investigate knee OA risk according to the change in obesity status over 2 years for subjects who had undergone health examinations for 2 consecutive years. General obesity without central obesity (HR 1.281, 95% CI 1.270–1.292) and central obesity without general obesity (HR 1.167, 95% CI 1.150–1.184) were associated with increased knee OA risk than the comparison group. Individuals with both general with central obesity had the highest risk (HR 1.418, 95% CI 1.406–1.429). This association was more pronounced in women and younger age group. Remarkably, the remission of general or central obesity over two years was associated with decreased knee OA risk (HR 0.884; 95% CI 0.867–0.902; HR 0.900; 95% CI 0.884–0.916, respectively). The present study found that both general and central obesity were associated with increased risk of knee OA and the risk was highest when the two types of obesity were accompanied. Changes in obesity status have been confirmed to alter the risk of knee OA.

## Introduction

Knee osteoarthritis (OA) is a common progressive multifactorial disorder^1^. It causes chronic pain, functional impairment, decreased quality of life, and economic burden^2^. The reported prevalence of OA in adults varies from 1.6 to 27.1%, depending on the definition of OA, study population and country. Recent systematic review and meta-analysis, including 88 studies, reported the global pooled prevalence of knee osteoarthritis over the age of 40 as 22.9% (95% CI 19.8–26.1%)^3^. It also shows sex and ethnic differences. Generally, women are affected more than men^4^. Additionally, although Asians are relatively thin, the knee OA prevalence in Asia is higher than that in Europe and North America^3^.

Knee OA accounts for nearly 80% of the international burden of OA^5^. In Korea, knee OA ranks fifth in the number of outpatients over the age of 60 (third among chronic diseases), and the total treatment cost of outpatient and inpatient care reached almost 1.14 billion dollars in 2020^6^. Obesity is an important risk factor in the development and progression of knee OA^7^. As the prevalence of obesity is steadily increasing worldwide^8,9^, the problem of increasing numbers of patients with knee OA is emerging.

The association between body mass index (BMI), a commonly used indicator of general obesity, and knee OA is also well known^4^. However, BMI makes it difficult to discriminate between body fat and lean mass or reveal central obesity^10,11^. Recently, waist circumference (WC) was reported to be a more appropriate indicator of central obesity-associated health risk than other obesity markers^11,12^. Asians are more likely to be centrally obese than Europeans with the same BMI^13,14^, and the difference in central fat mass can be significant even at the same BMI^15,16^. Previous studies have reported the relationship between knee OA and obesity based on only the BMI category^4,17–19^. Previous studies have evaluated the individual and combined effects of general and central obesity on knee OA. However, no nationwide studies have been conducted on the effect of obesity status and change on the risk of knee OA^48,49^. Understanding the association of obesity and changes in obesity status with risk of knee OA is important for developing efficient public health strategies.

The aim of this study was to evaluate the association between general and/or central obesity and the risk of knee OA in a nationwide population-based cohort study using the Korean National Health Insurance Service database. We further assessed how two-year changes in obesity status affected the risk of knee OA.

## Materials and methods

### Data and study population

The present study was conducted with retrospective cohort data obtained from the Korean National Health Insurance Service (KNHIS). The KNHIS is a government-run single insurance company with approximately 97% of the people subscribed. The cohort sample extracted through the KNHIS database represents the actual Korean population^20,21^. The KNHIS database includes enrollee anthropometric characteristics, medical institution use, disease codes identified by physicians, and medication prescription claims data^22–24^. The national medical screening program recommends that all enrollees over the age of 40 years undergo a general health examination at least once every two years^20,25,26^. Routine health examination obtains demographic data thorough standardized measurements and information on drinking, smoking, physical activity, and medical history through self-report surveys.

**Figure 1:**
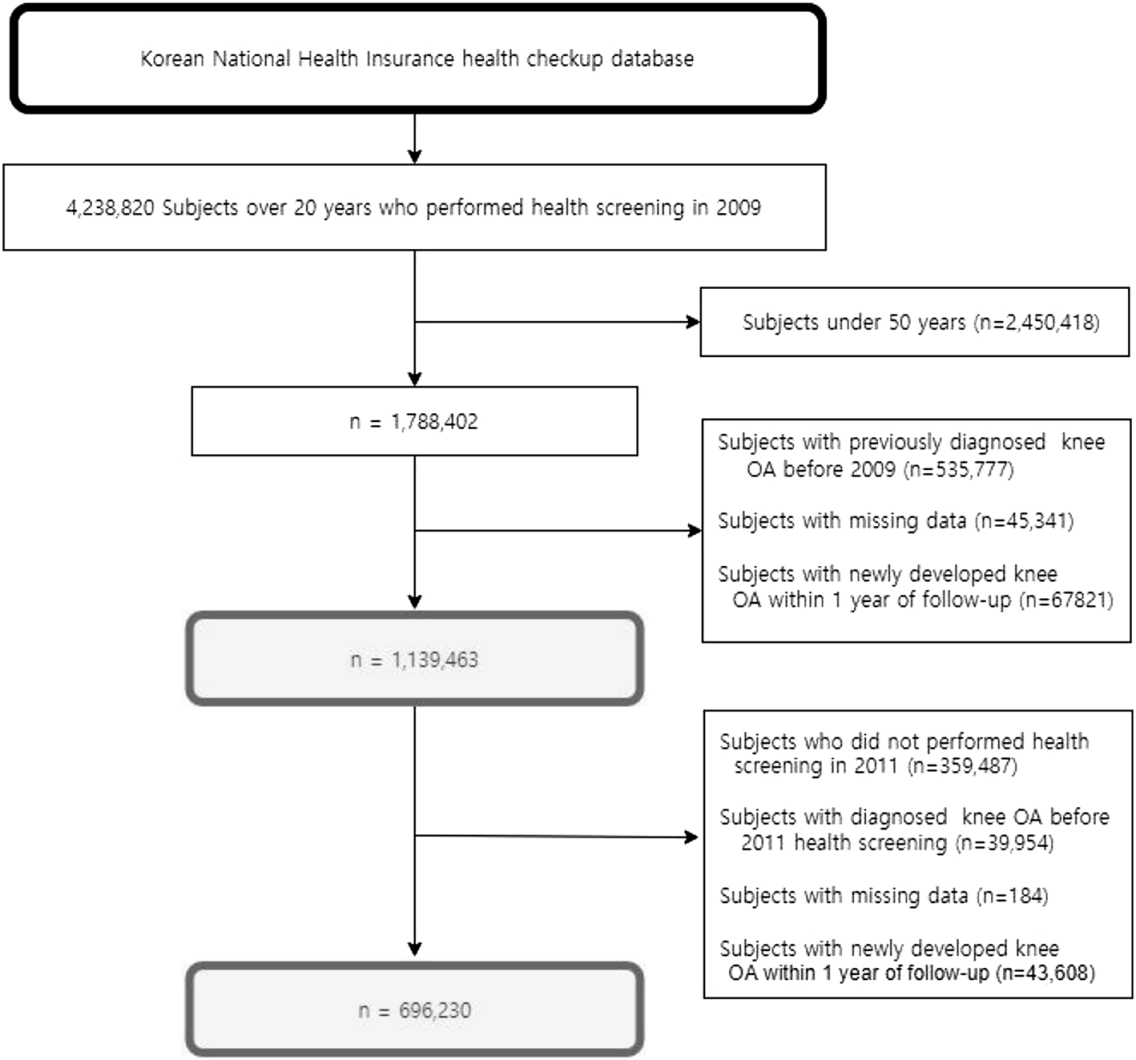
Flow chart of the cohort selection.

This nationwide retrospective cohort database includes a random sample of 1,788,402 people aged over 50 years at the index year of 2009. To minimize reversed causality and confounders, subjects diagnosed with knee OA prior to the index year and those with knee OA within one year were excluded. Subjects with missing values were also excluded. Finally, a retrospective analysis was conducted on 1,139,463 subjects (Fig. 1). The study cohort was followed up from the index date to the date of knee OA diagnosis or until 31 December 2018. The mean follow-up period was 6.53 ± 2.71 years.

### General health behaviors and comorbidities

Information on lifestyle-related factors was collected using the questionnaires. The categories of smoking status were as follows: never, former and current smoker. Drinking status was categorized as none, moderate (1–< 30 g/day), and heavy (≥ 30 g/day). Regular exercise was defined as when (1) moderate physical activity for > 30 min, ≥ 5 times/week or (2) vigorous physical activity for > 20 min, ≥ 3 times/week^27^. Income status was classified into quartiles based on the annual insurance premium to the KNHIS.

Comorbidities were defined per a previously validated method^28–31^. Hypertension, type 2 diabetes mellitus (T2DM) and dyslipidemia were defined using a diagnostic combination identified through the ICD-10 code and claims data of related drugs or the measured value of the health examination. Other comorbidities considered in this study included heart failure (HF), chronic obstructive pulmonary disease (COPD), chronic kidney disease (CKD), end-stage renal disease (ESRD), stroke, liver cirrhosis (LC), dementia, and cancer. Each definition is presented in Table S1. Blood collection was performed after fasting for at least 8 h from midnight to check the concentrations of glucose and creatine and determine the lipid profile.

### Definitions of general and central obesity

General obesity was defined as BMI ≥ 25 kg/m^2^, and BMI was calculated as the weight in kilograms divided by the square of the height in meters^11^. Following World Health Organization (WHO) recommendations for Asians, individuals were categorized as (1) underweight (BMI < 18.5 kg/m^2^), (2) normal (≥ 18.5 to < 23 kg/m^2^, reference group), (3) overweight (≥ 23 to < 25 kg/m^2^), (4) class 1 obese (≥ 25 to 30 kg/m^2^), and (5) class 2 obese (≥ 30 kg/m^2^)^32^.

WC was measured in the centerline between the rib cage and the iliac crest when the participant exhaled while standing by trained inspectors^33^. Regarding central obesity, subjects were grouped into 6 levels at 5 cm WC intervals as follows: (1) < 80 cm in men and < 75 cm in women, (2) 80–< 85 cm in men and 75–< 80 cm in women, (3) 85–< 90 cm in men and 80–< 85 cm in women (reference group), (4) 90–< 95 cm in men and 85–< 90 cm in women, (5) 95–< 100 cm in men and 90–< 95 cm in women, and (6) ≥ 100 cm in men and ≥ 95 cm in women. According to the definition of the Korean Society for the Study of obesity, central obesity was defined as a WC ≥ 90 cm in men and ≥ 85 cm in women^33^.

The change in obesity was evaluated as changes in obesity status between two years of the individuals who underwent routine health examination in both 2009 and 2011. Subjects were classified based on the presence of general or central obesity in the preceding and subsequent health examinations. This evaluation was conducted on 696,230 subjects by applying identical exclusion criteria for the study cohort (Fig. 1).

### Definition of knee OA

The primary endpoint was the newly developed knee OA. Based on a previous validation study, knee OA was defined by knee OA diagnostic code (M17) or any OA diagnostic code (M15, polyarthrosis or M19, other arthrosis) in combination with a procedure for a knee X-ray in the same claim^34^.

### Ethical considerations

The entire process of this study complied with the ethical norms of the Declaration of Helsinki. This study was approved by both the KNHIS and the IRB of the Catholic University of Korea (IRB No. VC22ZISI0014), and informed consent was exempted IRB of the Catholic University of Korea due to the anonymity of the data and the retrospective nature of the study.

### Statistical analysis

Continuous variables are presented as the means and standard deviations and were compared using the *t test* or ANOVA. Categorical variables are expressed as numbers and percentages and were compared by means of the chi-square test. Incidence rates were calculated by dividing the number of incident cases by the total number of person-years of follow-up and expressed as per 1,000 person-years. To evaluate the risk of knee OA associated with BMI and WC, we assessed the hazard ratios (HRs) and 95% confidence intervals (CIs) using Cox’s proportional hazards models. The variables used for adjustment from Model 1 to Model 4 are as follows: (model 1) adjusted for age and sex, (modle2) adjusted for model 1 plus income, smoking, drinking, exercise, (model 3) adjusted for model 2 plus hypertension, T2DM, dyslipidemia, (model 4) adjusted for model 3 plus ESRD, COPD, stroke, LC, HF, dementia, CKD and cancer. To assess the increasing category of obesity associated with risk of knee OA, P for linear trend was applied with linear regression, using the ordinal number assigned to each category of obesity. Stratified analyses according to sex and age were performed on the combination of general and central obesity and interaction tests were performed using a likelihood ratio test.

A *P* values provided are two-sided, with the level of significance at 0.05. All statistical analysis procedures were performed with SAS version 9.4 (SAS Institute, Cary, NC, USA).

## Results

### Baseline characteristics

The descriptive baseline characteristics of study populations are summarized in Table 1. Overall, 33.5% of subjects had general obesity (BMI ≥ 25 kg/m^2^, Table S2), and 46.9% had central obesity (WC ≥ 90 cm in males, ≥ 85 cm in females, Table S3). Among the study population, 403,050 (35.4%) developed knee OA during the follow-up period. The baseline mean BMI and WC of the knee OA group were 24.2 ± 2.9 kg/m^2^ and 82.1 ± 8.3 cm, respectively, and those of the non-OA group were 23.7 ± 2.9 kg/m^2^ and 81.8 ± 8.3 cm, respectively (both *P* < 0.0001). The knee OA group was more likely to be older and comprised more females, less smokers, less alcohol consumption, more hypertension, and more dyslipidemia than the non-OA group.

**Table 1:** Baseline characteristics of study participants according to knee OA development.

	Knee osteoarthritis		
	No (n = 736,413)	Yes (n = 403,050)	P-value
Age			
Mean	58.5 ± 7.7	59.9 ± 7.6	< 0.0001
Categories, n (%)			
50–59	460,080 (62.5)	212,070 (52.6)	< 0.0001
60–69	194,718 (26.4)	137,412 (34.1)	
70–79	70,034 (9.5)	48,661 (12.1)	
80–	11,581 (1.6)	4907 (1.2)	
Male, n (%)	483,669 (65.7)	179,481 (44.5)	< 0.0001
Low income < 25%, n (%)	166,502 (22.6)	95,279 (23.6)	< 0.0001
Type 2 DM, n (%)	109,443 (14.9)	55,838 (13.9)	< 0.0001
Hypertension, n (%)	302,508 (41.1)	179,064 (44.4)	< 0.0001
Dyslipidemia, n (%)	177,167 (24.1)	112,025 (27.8)	< 0.0001
Smoking, n (%)			
Non	401,912 (54.6)	278,925 (69.2)	< 0.0001
Ex	154,072 (20.9)	60,821 (15.1)	
Current	180,429 (24.5)	63,304 (15.7)	
Alcohol, n (%)			
Non	410,314 (55.7)	264,540 (65.6)	< 0.0001
Mild	265,261 (36.0)	112,450 (27.9)	
Heavy	60,838 (8.3)	26,060 (6.5)	
Regular exercise, n (%)	165,294 (22.5)	88,377 (21.9)	< 0.0001
Cancer, n (%)	15,865 (2.2)	8846 (2.2)	0.1569
ESRD, n (%)	789 (0.1)	318 (0.1)	< 0.0001
COPD, n (%)	46,233 (6.3)	33,449 (8.3)	< 0.0001
Stroke, n (%)	18,511 (2.5)	11,520 (2.9)	< 0.0001
LC, n (%)	3826 (0.5)	1511 (0.4)	< 0.0001
HF, n (%)	5620 (0.8)	3827 (1.0)	< 0.0001
Dementia, n (%)	5406 (0.7)	2848 (0.7)	0.0981
CKD, n (%)	63,877 (8.7)	37,821 (9.4)	< 0.0001
Height, cm	162.7 ± 8.2	159.8 ± 8.4	< 0.0001
Weight, kg	62.9 ± 10.2	62.0 ± 10.0	< 0.0001
BMI, kg/m^2^	23.7 ± 2.9	24.2 ± 2.9	< 0.0001
WC, cm	81.8 ± 8.3	82.1 ± 8.3	< 0.0001
Glucose, mg/dL	102.8 ± 28.8	101.3 ± 25.9	< 0.0001
SBP, mmHg	126.1 ± 15.8	126.0 ± 15.7	0.043
DBP, mmHg	78.1 ± 10.3	77.7 ± 10.1	< 0.0001
Total cholesterol, mg/dL	199.6 ± 37.8	201.4 ± 38.1	< 0.0001
HDL-C, mg/dL	54.8 ± 28.7	55.9 ± 31.5	< 0.0001
LDL-C, mg/dL	117.7 ± 39	119.3 ± 39.3	< 0.0001
Triglyceride^a^, mg/dL	121.8 (121.64–121.95)	119.87 (119.67–120.07)	< 0.0001
e-GFR, mL/min/1.73 m^2^	83.2 ± 35.2	83.2 ± 32.6	0.9422

Continuous variables were presented using mean and standard deviation. Categorical variables are expressed in numbers and percentages.

*DM* diabetes mellitus, *ESRD* end-stage renal disease, *COPD* chronic obstructive pulmonary disease, *LC* liver cirrhosis, *HF* hear failure, *CKD* chronic kidney disease, *BMI* body mass index, *WC* waist circumference, *SBP* systolic blood pressure, *DBP* diastolic blood pressure, *HDL* high-density lipoprotein cholesterol, *LDL-C* low-density lipoprotein cholesterol, *e-GFR* estimated glomerular filtration rate.

^a^Geometric mean values for triglyceride.

### Risk of knee OA according to general or central obesity

We assessed the risk of knee OA according to BMI and WC categories (Table 2). After adjustments for confounders, the risk of knee OA increased significantly with increasing BMI and WC, and a dose-dependent association was identified (both *P* for trend < 0.0001). The HRs of knee OA for general obesity and central obesity compared to comparison group were 1.337 (95% CI 1.328–1.345) and 1.289 (95% CI 1.280–1.298), respectively. Of note, compared to that of the comparison group, the risk of knee OA was 73.4% higher for groups with BMIs ≥ 30.0 kg/m^2^ and 30.1% higher for groups with WCs ≥ 100 cm in men and ≥ 95 cm in women, respectively.

**Table 2:** Incidence rate and hazard ratio for the risk of knee OA according to BMI and WC.

	IR (per 1000)	HR (95% CI)			
		Model 1	Model 2	Model 3	Model 4
BMI, kg/m^2^					
–< 18.5	38.85	0.749 (0.731, 0.767)	0.752 (0.734, 0.770)	0.753 (0.736, 0.772)	0.749 (0.732, 0.768)
18.5–< 23	47.59	1 (ref.)	1 (ref.)	1 (ref.)	1 (ref.)
23–< 25	53.88	1.204 (1.194, 1.213)	1.202 (1.192, 1.212)	1.200 (1.190, 1.209)	1.201 (1.191, 1.211)
25–< 30	61.57	1.424 (1.413, 1.435)	1.419 (1.409, 1.430)	1.415 (1.404, 1.426)	1.417 (1.406, 1.428)
30–	81.16	1.747 (1.716, 1.778)	1.740 (1.709, 1.771)	1.734 (1.703, 1.766)	1.734 (1.703, 1.766)
*P* for trend		< 0.0001	< 0.0001	< 0.0001	< 0.0001
BMI, kg/m^2^					
–< 25	49.89	1 (ref.)	1 (ref.)	1 (ref.)	1 (ref.)
25–	62.91	1.350 (1.342, 1.359)	1.345 (1.337, 1.354)	1.335 (1.327, 1.344)	1.337 (1.328, 1.345)
*P* value		< 0.0001	< 0.0001	< 0.0001	< 0.0001
WC, cm (male/female)					
− < 80/− < 75	46.36	0.765 (0.758, 0.771)	0.767 (0.760, 0.774)	0.770 (0.763, 0.776)	0.769 (0.762, 0.776)
− < 85/− < 80	50.89	0.902 (0.894, 0.910)	0.902 (0.894, 0.910)	0.903 (0.896, 0.911)	0.904 (0.896, 0.912)
− < 90/− < 85	56.36	1 (ref.)	1 (ref.)	1 (ref.)	1 (ref.)
− < 95/− < 90	61.57	1.103 (1.092, 1.114)	1.102 (1.091, 1.113)	1.101 (1.090, 1.112)	1.101 (1.090, 1.112)
− < 100/− < 95	69.18	1.193 (1.177, 1.208)	1.191 (1.175, 1.206)	1.190 (1.174, 1.205)	1.189 (1.173, 1.205)
100–/95–	79.75	1.304 (1.282, 1.327)	1.303 (1.280, 1.326)	1.302 (1.280, 1.325)	1.301 (1.278, 1.324)
*P* for trend		< 0.0001	< 0.0001	< 0.0001	< 0.0001
WC, cm (male/female)					
− < 90/− < 85	50.93	1 (ref.)	1 (ref.)	1 (ref.)	1 (ref.)
90–/85–	65.53	1.306 (1.297, 1.315)	1.302 (1.292, 1.311)	1.289 (1.280, 1.299)	1.289 (1.280, 1.298)
*P* value		< 0.0001	< 0.0001	< 0.0001	< 0.0001

Model 1 was adjusted for age, sex. Model 2 was adjusted for age, sex, income, smoking, alcohol intake and regular exercises. Model 3 was adjusted for age, sex, income, smoking, alcohol intake, regular exercises, diabetes, hypertension and dyslipidemia. Model 4 was adjusted for age, sex, income, smoking, alcohol intake, regular exercises, diabetes, hypertension, dyslipidemia, cancer, end-stage renal disease, chronic obstructive pulmonary disease, stroke, liver cirrhosis, hear failure, dementia and chronic kidney disease.

*BMI* body mass index, *WC* waist circumference, *HR* hazard ratio, *CI* confidence interval, *OA* osteoarthritis, *IR* incidence rate.

**Figure 2:**
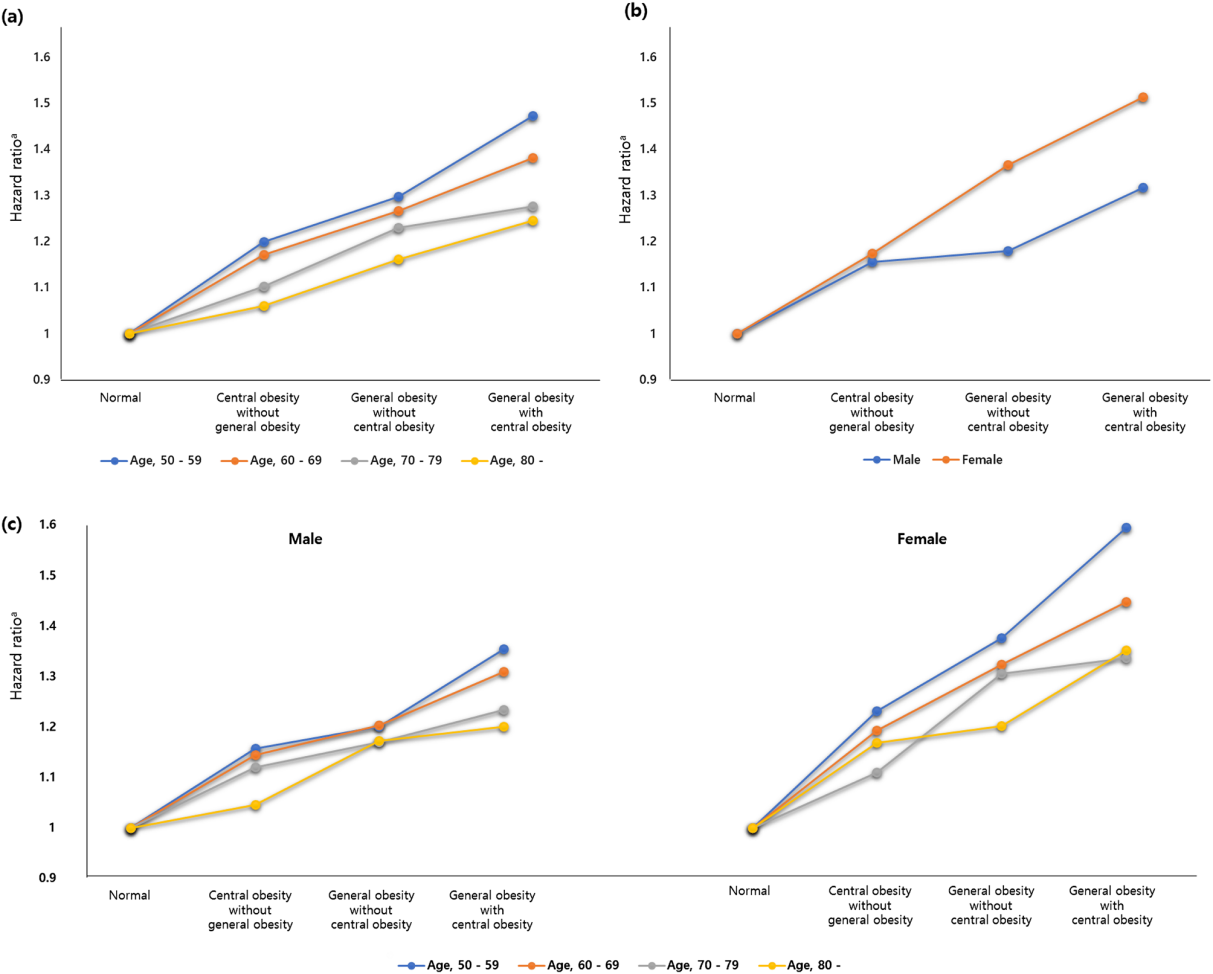
Adjusted knee OA risk for general and/or central obesity composition, according to (**a**) age, (**b**) sex and (**c**) age and sex. ^a^Adjusted for age, sex, income, smoking, alcohol intake, regular exercises, diabetes, hypertension, dyslipidemia, cancer, end-stage renal disease, chronic obstructive pulmonary disease, stroke, liver cirrhosis, hear failure, dementia and chronic kidney disease.

In the analysis of knee OA risk according to general and/or central obesity, the highest risk was identified in those who had both general with central obesity (HR 1.418; 95% CI 1.406–1.429) (Table 3). Even having only central obesity without general obesity increased the risk (HR 1.167; 95% CI 1.150–1.184) (Table 3). In the subgroup analysis, females (HR 1.513; 95% CI 1.496–1.529) were more associated with knee OA risk than males (HR 1.317; 95% CI 1.302–1.332) when accompanied by general and central obesity (Table 4, Fig. 2). It has been found that younger age groups have a higher HR of knee OA associated with general and/or central obesity (Table 4, Fig. 2).

**Table 3:** Incidence rate and hazard ratio for the risk of knee OA according to general obesity and/or central obesity composition.

General obesity	Central obesity	IR (per 1000)	HR (95% CI)			
			Model 1	Model 2	Model 3	Model 4
No	No	49.06	1 (ref.)	1 (ref.)	1 (ref.)	1 (ref.)
	Yes	61.49	1.173 (1.156, 1.190)	1.171 (1.154, 1.188)	1.168 (1.152, 1.185)	1.167 (1.150, 1.184)
Yes	No	58.69	1.290 (1.279, 1.301)	1.286 (1.275, 1.297)	1.279 (1.268, 1.290)	1.281 (1.270, 1.292)
	Yes	66.58	1.432 (1.421, 1.443)	1.426 (1.415, 1.437)	1.417 (1.406, 1.429)	1.418 (1.406, 1.429)

Model 1 was adjusted for age, sex. Model 2 was adjusted for age, sex, income, smoking, alcohol intake and regular exercises. Model 3 was adjusted for age, sex, income, smoking, alcohol intake, regular exercises, diabetes, hypertension and dyslipidemia. Model 4 was adjusted for age, sex, income, smoking, alcohol intake, regular exercises, diabetes, hypertension, dyslipidemia, cancer, end-stage renal disease, chronic obstructive pulmonary disease, stroke, liver cirrhosis, hear failure, dementia and chronic kidney disease.

*HR* hazard ratio, *CI* confidence interval, *OA* osteoarthritis, *IR* incidence rate.

**Table 4:** Incidence rate and hazard ratio for the risk of knee OA of the general obesity and/or central obesity composition according to age and sex.

General obesity	Central obesity	IR (per 1000)	HR (95% CI)			
			Model 1	Model 2	Model 3	Model 4
Age categories						
50–59					P for interaction < 0.0001	
No	No	41.70	1 (ref.)	1 (ref.)	1 (ref.)	1 (ref.)
	Yes	47.74	1.204 (1.176, 1.232)	1.202 (1.174, 1.230)	1.200 (1.172, 1.228)	1.198 (1.170, 1.227)
Yes	No	50.79	1.304 (1.290, 1.319)	1.300 (1.286, 1.315)	1.295 (1.280, 1.310)	1.296 (1.281, 1.311)
	Yes	55.56	1.484 (1.468, 1.501)	1.477 (1.461, 1.494)	1.470 (1.454, 1.487)	1.470 (1.453, 1.486)
60–69						
No	No	59.69	1 (ref.)	1 (ref.)	1 (ref.)	1 (ref.)
	Yes	71.34	1.175 (1.149, 1.202)	1.173 (1.147, 1.200)	1.172 (1.146, 1.199)	1.170 (1.143, 1.196)
Yes	No	75.20	1.271 (1.252, 1.291)	1.268 (1.249, 1.288)	1.263 (1.244, 1.282)	1.265 (1.246, 1.285)
	Yes	81.24	1.389 (1.371, 1.407)	1.384 (1.366, 1.402)	1.379 (1.361, 1.397)	1.379 (1.361, 1.398)
70–79						
No	No	66.63	1 (ref.)	1 (ref.)	1 (ref.)	1 (ref.)
	Yes	77.83	1.105 (1.070, 1.141)	1.103 (1.069, 1.139)	1.103 (1.068, 1.139)	1.102 (1.067, 1.137)
Yes	No	83.07	1.230 (1.194, 1.267)	1.227 (1.191, 1.265)	1.223 (1.187, 1.260)	1.229 (1.193, 1.267)
	Yes	87.72	1.278 (1.250, 1.307)	1.275 (1.247, 1.304)	1.272 (1.244, 1.301)	1.275 (1.247, 1.304)
80–						
No	No	58.94	1 (ref.)	1 (ref.)	1 (ref.)	1 (ref.)
	Yes	67.25	1.061 (0.972, 1.159)	1.060 (0.971, 1.157)	1.060 (0.971, 1.158)	1.061 (0.972, 1.159)
Yes	No	70.02	1.169 (1.038, 1.317)	1.165 (1.035, 1.313)	1.161 (1.031, 1.308)	1.161 (1.031, 1.308)
	Yes	76.06	1.253 (1.16, 1.355)	1.25 (1.157, 1.352)	1.248 (1.154, 1.349)	1.244 (1.151, 1.345)
Sex category						
Male					P for interaction < 0.0001	
No	No	36.08	1 (ref.)	1 (ref.)	1 (ref.)	1 (ref.)
	Yes	44.74	1.168 (1.144, 1.193)	1.163 (1.138, 1.187)	1.160 (1.136, 1.185)	1.156 (1.132, 1.181)
Yes	No	40.40	1.188 (1.172, 1.204)	1.180 (1.165, 1.196)	1.174 (1.159, 1.190)	1.179 (1.164, 1.195)
	Yes	47.30	1.335 (1.320, 1.351)	1.323 (1.308, 1.338)	1.315 (1.300, 1.330)	1.317 (1.302, 1.332)
Female						
No	No	67.38	1 (ref.)	1 (ref.)	1 (ref.)	1 (ref.)
	Yes	90.01	1.175 (1.153, 1.198)	1.177 (1.154, 1.200)	1.174 (1.151, 1.197)	1.174 (1.151, 1.197)
Yes	No	91.13	1.376 (1.360, 1.392)	1.374 (1.359, 1.390)	1.367 (1.351, 1.383)	1.366 (1.350, 1.382)
	Yes	108.14	1.523 (1.506, 1.539)	1.523 (1.507, 1.540)	1.514 (1.497, 1.530)	1.513 (1.496, 1.529)

Model 1 was adjusted for age, sex. Model 2 was adjusted for age, sex, income, smoking, alcohol intake and regular exercises. Model 3 was adjusted for age, sex, income, smoking, alcohol intake, regular exercises, diabetes, hypertension and dyslipidemia. Model 4 was adjusted for age, sex, income, smoking, alcohol intake, regular exercises, diabetes, hypertension, dyslipidemia, cancer, end-stage renal disease, chronic obstructive pulmonary disease, stroke, liver cirrhosis, hear failure, dementia and chronic kidney disease.

*HR* hazard ratio, *CI* confidence interval, *OA* osteoarthritis, *IR* incidence rate.

### Risk of knee OA according to changes in obesity status

Compared to those without general and central obesity over two years, the HRs of knee OA in those who developed obesity in the subsequent health examinations were 1.229 (95% CI 1.206–1.253) for general obesity and 1.226 (95% CI 1.207–1.245) for central obesity (Table 5). Remarkably, those whose general or central obese status was resolved still had a higher risk of knee OA (HR 1.216; 95% CI 1.193–1.239; HR 1.227; 95% CI 1.208–1.247, respectively). On the other hand, compared to those whose general or central obesity remained, the knee OA risks in those whose obesity status was resolved were significantly reduced by 11.6% for general obesity and 10.0% for central obesity (Table 6).

**Table 5:** Incidence rate and hazard ratio for the risk of knee OA according to the change in obesity status.

Change in obesity (pre/post)	IR (per 1000)	HR (95% CI)			
		Model 1	Model 2	Model 3	Model 4
General obesity					
No/no	50.63	1 (ref.)	1 (ref.)	1 (ref.)	1 (ref.)
No/yes	59.42	1.236 (1.213, 1.26)	1.235 (1.212, 1.259)	1.230 (1.207, 1.254)	1.229 (1.206, 1.253)
Yes/no	59.57	1.222 (1.199, 1.246)	1.220 (1.197, 1.243)	1.215 (1.192, 1.238)	1.216 (1.193, 1.239)
Yes/yes	64.46	1.388 (1.374, 1.401)	1.383 (1.369, 1.396)	1.374 (1.360, 1.388)	1.375 (1.361, 1.389)
Central obesity					
No/no	51.17	1 (ref.)	1 (ref.)	1 (ref.)	1 (ref.)
No/yes	63.55	1.235 (1.216, 1.254)	1.233 (1.214, 1.252)	1.226 (1.208, 1.246)	1.226 (1.207, 1.245)
Yes/no	63.21	1.237 (1.217, 1.256)	1.233 (1.214, 1.253)	1.227 (1.208, 1.247)	1.227 (1.208, 1.247)
Yes/yes	67.76	1.382 (1.365, 1.398)	1.376 (1.359, 1.392)	1.365 (1.348, 1.382)	1.363 (1.347, 1.380)

Model 1 was adjusted for age, sex. Model 2 was adjusted for age, sex, income, smoking, alcohol intake and regular exercises. Model 3 was adjusted for age, sex, income, smoking, alcohol intake, regular exercises, diabetes, hypertension and dyslipidemia. Model 4 was adjusted for age, sex, income, smoking, alcohol intake, regular exercises, diabetes, hypertension, dyslipidemia, cancer, end-stage renal disease, chronic obstructive pulmonary disease, stroke, liver cirrhosis, hear failure, dementia and chronic kidney disease.

(Pre/post) means obesity status in the preceding and subsequent health examination.

*HR* hazard ratio, *CI* confidence interval, *OA* osteoarthritis, *IR* incidence rate.

**Table 6:** Change in hazard ratio for the risk of knee OA due to the resolution of obesity in obese subjects.

Change in obesity (pre/post)	HR (95% CI)			
	Model 1	Model 2	Model 3	Model 4
General obesity				
Yes/yes	1 (ref.)	1 (ref.)	1 (ref.)	1 (ref.)
Yes/no	0.881 (0.864, 0.898)	0.882 (0.865, 0.899)	0.884 (0.867, 0.902)	0.884 (0.867, 0.902)
Central obesity				
Yes/yes	1 (ref.)	1 (ref.)	1 (ref.)	1 (ref.)
Yes/no	0.895 (0.879, 0.912)	0.897 (0.880, 0.913)	0.899 (0.883, 0.916)	0.900 (0.884, 0.916)

Model 1 was adjusted for age, sex. Model 2 was adjusted for age, sex, income, smoking, alcohol intake and regular exercises. Model 3 was adjusted for age, sex, income, smoking, alcohol intake, regular exercises, diabetes, hypertension and dyslipidemia. Model 4 was adjusted for age, sex, income, smoking, alcohol intake, regular exercises, diabetes, hypertension, dyslipidemia, cancer, end-stage renal disease, chronic obstructive pulmonary disease, stroke, liver cirrhosis, hear failure, dementia and chronic kidney disease.

(Pre/post) means obesity status in the preceding and subsequent health examination.

*HR* hazard ratio, *CI* confidence interval, *OA* osteoarthritis, *IR* incidence rate.

## Discussion

In this nationwide population-based retrospective cohort study of 1,139,463 participants aged 50 and older, we found that (1) Higher BMI and greater WC increase the risk of knee OA in a dose-dependent manner; (2) Those who had both general and central obesity had the highest risk of knee OA and its associations were stronger in women and younger people; and (3) Changes in obesity status altered the risk of knee OA. In particular, the risk was reduced in the obesity-resolved group compared to the obesity-maintaining group.

Although obesity has been reported to be an important risk factor for knee OA, to the best of our knowledge, this is the first national cohort study to determine the risk of knee OA in Asian people with general and central obesity. General obesity is defined on the basis of BMI, a standard anthropometric parameter, and high BMI is one of the strongest risk factors for knee OA. General obesity causes greater mechanical stress due to excessive weight on the joint surface, which leads to cartilage degeneration and OA. Indeed, with a weight gain of 1 kg, there is a six-fold increase in on both sides of the knee^35^. A meta-analysis of 22 cohort and patient-control studies reported that the pooled odds ratio of knee OA for overweight-to-normal BMI was 2.18 (95% CI 1.86–2.55), and that for obese-to-normal BMI was 2.63 (95% CI 2.28–3.05)^36^. In a prospective cohort study of 105,189 patients with newly developed knee OA, obese patients had more than twice the risk of knee arthroplasty surgery than normal-weight patients^37^. The risk of general obesity for knee OA identified in our study is also in line with previous studies.

Traditionally, the relationship between obesity and OA has been understood in terms of mechanical load, but OA in joints that are not affected by weight, such as the hands, has also been confirmed to be related to obesity^38^. It is presumed to be related to cytokines secreted by adipose tissues (i.e., adipokines) that regulate bone and cartilage homeostasis^39–42^. Among those adipokines, leptin has been identified as a major degradation factor of cartilage and a mediator for OA. Cartilage cells have leptin receptors, and high concentrations of leptin are associated with the development and progression of OA^43^. The leptin concentration was found to be higher in the synovial fluid of the osteoarthritic joint than in normal tissue^44^. The present study supports the hypothesis about the role of regional adipose tissue in OA by confirming the independent association between central obesity and the risk of knee OA. Therefore, it is inferred that the composition of general and central obesity increases the risk of knee OA due to the synergy of mechanical stress and the degradation-mediated reaction of adipose tissue.

Our findings showed that the association between obesity and risk of knee OA was stronger in female. Although the mechanism by which sex differences affect the development of knee OA is still unclear, various factors have been suggested as possible causes. First, the normal knee joint cartilage volume of males is significantly larger than that of females^45^. In a study using MRI to determine changes in knee articular cartilage defects over a 2.3-year period among 211 participants without clinical OA, females had a threefold higher risk of developing knee articular cartilage defects than males, even among healthy participants^45^. In addition, hormonal differences between males and females are also suggested to play an important role. In particular, postmenopausal estrogen reduction in females is significantly associated with an increased risk of developing knee OA^46^. The presence of estrogen receptors identified in joint cartilage suggests a relationship between female hormones and joint cartilage^47^.

Present study confirms that younger group with obesity are more susceptible to knee OA. Several studies have reported an association between general obesity and knee osteoarthritis^50,51^, and the metabolic effects of central obesity are also known to be associated with osteoarthritis^52^. Therefore, considering the cumulative effects of both types of obesity on knee joint, obesity at an early age may be associated with a greater risk than a later age.

Identifying the risk of obesity and groups more vulnerable to its harm is useful for focusing management strategies. This study highlights the stronger association between obesity and knee OA in women and younger populations. From a public health perspective, just as screening for osteoporosis is provided to postmenopausal women in their 50 s, more attention should be sought to prevent knee OA for obese women of a similar age.

In this study, changes in obesity status altered the risk of developing OA. The fact that resolution of general and central obesity in two years reduced the risk of developing knee OA by 11.6% and 10.0%, respectively, is promising evidence for the importance of intensive interventions in obese populations. It is noteworthy that baseline normal subjects who developed new general obesity had a higher risk of knee OA than baseline general obese subjects who achieved remission of obesity for 2 years in the population aged 50 years and older (Table 5). These findings further emphasize the importance of obesity management and provide a high level of motivation for behavioral changes toward a healthy lifestyle.

The present study has several limitations. First, since the disease was defined based on the claims data in the NHIS database, the potential for bias due to misclassification cannot be overlooked, which might lead to the possibility of underestimation or overestimation. In addition, because our study was based on claims data, we could not confirm information on the grade or treatment of knee OA. Second, the change in obesity was evaluated only as a result of two years, and the cause of the change could not be identified. Third, a selection bias may exist between enrolled and missing participants for the analysis of changes in obesity status. Due to the large sample size, the difference in obesity and comorbidity between the two groups has statistically significant, but the magnitude of the difference was not large. Details are shown in Table S7. Fourth, drug use or eating habits that may affect weight were not considered. Fifth, medical history were obtained through a self-reported questionnaire. This suggests that there might be some bias that could affect the results after adjustments. Sixth, since our research was conducted with the data of the Korean NHIS, there may be limitations in generalizing our research results to other ethnic groups.

Nevertheless, our study has the strength of a nationwide population-based cohort study. The KNHIS database provides large-scale health data (big data), which is rare worldwide. In addition, this study enhances the reliability and universality of the findings by adjusting for a wide range of comorbidities. To the best of our knowledge, this is the first nationwide study to confirm that a two-year change in obesity status decreases the risk of knee OA.

Obesity and knee OA are strongly associated with personal and social burdens. Therefore, our findings support further investigation of the relationship between obesity and knee OA for the establishment of more effective OA prevention strategies.

In conclusion, our nationwide retrospective cohort study confirmed that higher BMI and WC were associated with an increased risk of knee OA in a dose-dependent manner. Accompanying general obesity and central obesity increase the risk more, and these effects are stronger in women and younger age groups. Changes in obesity status have been confirmed to alter the risk of knee OA.

## Supplementary Information

Supplementary Information 1.

Supplementary Information 2.

## Data Availability

The data presented in this study are available in the main article.
