# Supplementary material for: Association of general and central obesity, and their changes with risk of knee osteoarthritis: a nationwide population-based cohort study
Source: Sci Rep. 2023 Mar 7;13:3796. doi: 10.1038/s41598-023-30727-4 (PMC9992488; doi:10.1038/s41598-023-30727-4)
Supplement: Supplementary file 1 — Supplementary Information 1. [file 41598_2023_30727_MOESM1_ESM.docx]

| **S1 Table. Definition of comorbidities** | | |
| --- | --- | --- |
| **Comorbidities** |  | **ICD-10-CM code and definition** |
| Hypertension ^a^ | | I10-I13, I15 and minimum one prescription of anti-hypertensive drug (thiazide, loop diuretics, aldosterone antagonist, alpha-/beta-blocker, calcium-channel blocker, angiotensin-converting enzyme inhibitor, and angiotensin II receptor blocker) ; or systolic BP (SBP) ≥ 140 mmHg or diastolic BP (DBP) ≥ 90 mmHg |
|  |  |  |
| Type 2 diabetes mellitus ^a^ | | E11-E14 and minimum one prescription of anti-diabetic drugs (sulfonylureas, metformin, meglitinides, thiazolidinediones, dipeptidyl peptidase-4 inhibitors, α-glucosidase inhibitors, and insulin) ; or fasting plasma glucose value ≥126 mg/d |
|  |  |  |
| Dyslipidemia ^a^ | | E78 and use of lipid-lowering agents ; or fasting total cholesterol ≥ 240 mg/dL |
|  |  |  |
| Heart failure ^b^ |  | I50 |
|  |  |  |
| Chronic obstructive pulmonary disease ^b^ |  | J41, J42, J43 and J44 |
|  |  |  |
| Chronic kidney disease |  | estimated GFR < 60 mL/min/1.73 m^2^ |
|  |  |  |
| End-stage renal disease ^b^ |  | V001, V003, V005 |
|  |  |  |
| Stroke ^b^ |  | I63 and I64 |
|  |  |  |
| Liver cirrhosis ^b^ | | K703 and K746 |
|  |  |  |
| Dementia ^b^ |  | F00, G30, F01, F02, F03, G23.1, G31.0, G31.1, G31.82, G31.83, G31.88, and F10.7 |
|  |  |  |
| Cancer ^b^ |  | C00-96 and V193 |
|  |  |  |
|  |  |  |

ICD-10-CM=the International Classification of Disease

^a^ Defined by combination of diagnosis identified through ICD-10 code (≥ 1 diagnosis during hospitalization or at the outpatient clinic, in the previous one year) and claim data of related drugs or measured value of health examination

^b^ Defined as ≥ 1 diagnosis during hospitalization or at the outpatient clinic, in the previous one year

**S2 Table. Baseline characteristics of study participants according to BMI category**

|  | **BMI category** | | | | | |
| --- | --- | --- | --- | --- | --- | --- |
|  | - <18.5 | 18.5 - <23 | 23 - <25 | 25 - <30 | 30 - | P-value |
|  | (n=28,757) | (n=411,948) | (n=317,387) | (n=353,369) | (n=28,002) |  |
| **Age** |  |  |  |  |  |  |
| mean | 63.18 ± 9.95 | 59.31 ± 8.12 | 58.77 ± 7.41 | 58.57 ± 7.16 | 58.16 ± 7.05 | <.0001 |
| Categories, n (%) |  |  |  |  |  |  |
| 50 - 59 | 12,080 (42.01) | 239,197 (58.06) | 189,731 (59.78) | 213,489 (60.42) | 17,653 (63.04) | <.0001 |
| 60 - 69 | 8,318 (28.93) | 115,896 (28.13) | 93,874 (29.58) | 106,123 (30.03) | 7,919 (28.28) |  |
| 70 - 79 | 6,421 (22.33) | 48,685 (11.82) | 30,384 (9.57) | 30,986 (8.77) | 2,219 (7.92) |  |
| 80 - | 1,938 (6.74) | 8,170 (1.98) | 3,398 (1.07) | 2,771 (0.78) | 211 (0.75) |  |
| **Male, n (%)** | 16,801 (58.42) | 222,498 (54.01) | 188,764 (59.47) | 221,019 (62.55) | 14,068 (50.24) | <.0001 |
| **Low income** <**25%, n (%)** | 7,073 (24.6) | 97,529 (23.68) | 71,176 (22.43) | 79,398 (22.47) | 6,605 (23.59) | <.0001 |
| **Type 2 DM, n (%)** | 2,700 (9.39) | 45,900 (11.14) | 45,266 (14.26) | 64,023 (18.12) | 7,392 (26.4) | <.0001 |
| **Hypertension, n (%)** | 7,933 (27.59) | 134,818 (32.73) | 133,385 (42.03) | 186,205 (52.69) | 19,231 (68.68) | <.0001 |
| **Dyslipidemia, n (%)** | 3,503 (12.18) | 82,723 (20.08) | 82,928 (26.13) | 109,203 (30.9) | 10,835 (38.69) | <.0001 |
| **Smoking, n (%)** |  |  |  |  |  | <.0001 |
| Non | 15,955 (55.48) | 252,646 (61.33) | 188,857 (59.5) | 205,058 (58.03) | 18,321 (65.43) |  |
| Ex | 3,600 (12.52) | 63,308 (15.37) | 63,535 (20.02) | 79,362 (22.46) | 5,088 (18.17) |  |
| Current | 9,202 (32) | 95,994 (23.3) | 64,995 (20.48) | 68,949 (19.51) | 4,593 (16.4) |  |
| **Alcohol, n (%)** |  |  |  |  |  | <.0001 |
| Non | 18,809 (65.41) | 254,235 (61.72) | 184,757 (58.21) | 199,400 (56.43) | 17,653 (63.04) |  |
| Mild | 7,862 (27.34) | 129,320 (31.39) | 109,293 (34.44) | 123,335 (34.9) | 7,901 (28.22) |  |
| Heavy | 2,086 (7.25) | 28,393 (6.89) | 23,337 (7.35) | 30,634 (8.67) | 2,448 (8.74) |  |
| **Regular exercise, n (%)** | 4,484 (15.59) | 86,942 (21.11) | 74,271 (23.4) | 82,397 (23.32) | 5,577 (19.92) | <.0001 |
| **Cancer, n (%)** | 997 (3.47) | 10,294 (2.5) | 6,453 (2.03) | 6,472 (1.83) | 495 (1.77) | <.0001 |
| **ESRD, n (%)** | 40 (0.14) | 522 (0.13) | 270 (0.09) | 252 (0.07) | 23 (0.08) | <.0001 |
| **COPD, n (%)** | 3,329 (11.58) | 29,232 (7.1) | 21,231 (6.69) | 23,816 (6.74) | 2,074 (7.41) | <.0001 |
| **Stroke, n (%)** | 841 (2.92) | 9,550 (2.32) | 8,317 (2.62) | 10,357 (2.93) | 966 (3.45) | <.0001 |
| **LC, n (%)** | 152 (0.53) | 1,961 (0.48) | 1,479 (0.47) | 1,600 (0.45) | 145 (0.52) | 0.1916 |
| **HF, n (%)** | 386 (1.34) | 3,002 (0.73) | 2,369 (0.75) | 3,278 (0.93) | 412 (1.47) | <.0001 |
| **Dementia, n (%)** | 562 (1.95) | 3,313 (0.8) | 1,993 (0.63) | 2,199 (0.62) | 187 (0.67) | <.0001 |
| **CKD, n (%)** | 2,496 (8.68) | 33,369 (8.1) | 28,252 (8.9) | 34,465 (9.75) | 3,116 (11.13) | <.0001 |
| **Height, cm** | 160.8 ± 8.76 | 161.17 ± 8.22 | 161.91 ± 8.35 | 162.23 ± 8.53 | 160.27 ± 9.18 | <.0001 |
| **Weight, kg** | 45.33 ± 5.48 | 55.49 ± 6.45 | 62.93 ± 6.66 | 70.29 ± 8 | 81.47 ± 9.68 | <.0001 |
| **BMI, kg/m^2^** | 17.48 ± 0.88 | 21.31 ± 1.16 | 23.93 ± 0.57 | 26.64 ± 1.25 | 31.65 ± 1.96 | <.0001 |
| **WC, cm** | 68.91 ± 6.12 | 76.4 ± 6.07 | 82.29 ± 5.63 | 87.92 ± 6.19 | 96.83 ± 7.57 | <.0001 |
| **Glucose, mg/dL** | 98.38 ± 30.1 | 99.56 ± 26.84 | 102.23 ± 27.2 | 105.14 ± 28.51 | 110.2 ± 31.86 | <.0001 |
| **SBP, mmHg** | 120.93 ± 16.97 | 123.36 ± 15.8 | 126.23 ± 15.28 | 128.95 ± 15.26 | 132.77 ± 15.89 | <.0001 |
| **DBP, mmHg** | 74.84 ± 10.53 | 76.24 ± 10.14 | 78.05 ± 9.97 | 79.79 ± 10.04 | 82.09 ± 10.35 | <.0001 |
| **Total cholesterol, mg/dL** | 188.08 ± 36.44 | 197.58 ± 37.04 | 201.49 ± 37.89 | 202.84 ± 38.44 | 204.92 ± 40.13 | <.0001 |
| **HDL-C, mg/dL** | 62.37 ± 36.83 | 57.9 ± 30.86 | 54.34 ± 28.51 | 52.49 ± 28.49 | 51.97 ± 27.38 | <.0001 |
| **LDL-C, mg/dL** | 107.66 ± 39.67 | 116.73 ± 38.22 | 119.67 ± 38.63 | 119.41 ± 39.95 | 119.92 ± 43.01 | <.0001 |
| **Triglyceride^a^, mg/dL** | 90.17 (89.67 - 90.68) | 105.74 (105.57 - 105.91) | 124.09 (123.86 - 124.32) | 139.75 (139.5 - 140) | 150.63 (149.72 - 151.56) | <.0001 |
| **e-GFR, mL/min/1.73 m^2^** | 86.49 ± 34.34 | 84.45 ± 34.04 | 82.75 ± 34.27 | 81.84 ± 34.48 | 81.69 ± 34.35 | <.0001 |

Continuous variables were presented using mean and standard deviation. Categorical variables are expressed in numbers and percentages

^a^Geometric mean values for triglyceride

Abbreviations: DM, diabetes mellitus; ESRD, end-stage renal disease; COPD, chronic obstructive pulmonary disease; LC, liver cirrhosis; HF, hear failure; CKD, chronic kidney disease; BMI, body mass index; WC, waist circumference; SBP, systolic blood pressure; DBP, diastolic blood pressure; HDL, high-density lipoprotein cholesterol; LDL-C, low-density lipoprotein cholesterol; e-GFR, estimated glomerular filtration rate

**S3 Table. Baseline characteristics of study participants according to WC category**

|  | **WC category** | | | | | | |  |
| --- | --- | --- | --- | --- | --- | --- | --- | --- |
|  | - <80 / - <75 | - <85 / - <80 | - <90 / - <85 | - <95 / - <90 | - <100 / - <95 | 100 - / 95 - | P-value | |
|  | (n=311826) | (n=293450) | (n=272413) | (n=162938) | (n=67712) | (n=31124) |  |  |
| **Age** |  |  |  |  |  |  |  | |
| mean | 58.44 ± 7.93 | 58.6 ± 7.48 | 59.13 ± 7.52 | 59.74 ± 7.65 | 60.27 ± 7.84 | 60.72 ± 8.13 | <.0001 | |
| Categories, n (%) |  |  |  |  |  |  |  | |
| 50 - 59 | 196,812 (63.12) | 179,701 (61.24) | 157,367 (57.77) | 88,172 (54.11) | 34,724 (51.28) | 15,374 (49.4) | <.0001 | |
| 60 - 69 | 78,374 (25.13) | 82,816 (28.22) | 83,991 (30.83) | 53,538 (32.86) | 22,933 (33.87) | 10,478 (33.67) |  | |
| 70 - 79 | 31,159 (9.99) | 27,318 (9.31) | 27,640 (10.15) | 19,020 (11.67) | 8,973 (13.25) | 4,585 (14.73) |  | |
| 80 - | 5,481 (1.76) | 3,615 (1.23) | 3,415 (1.25) | 2,208 (1.36) | 1,082 (1.6) | 687 (2.21) |  | |
| **Male, n (%)** | 155,586 (49.9) | 175,934 (59.95) | 167,843 (61.61) | 105,161 (64.54) | 41,378 (61.11) | 17,248 (55.42) | <.0001 | |
| **Low income** <**25%, n (%)** | 74,466 (23.88) | 66,391 (22.62) | 61,196 (22.46) | 36,927 (22.66) | 15,605 (23.05) | 7,196 (23.12) | <.0001 | |
| **Type 2 DM, n (%)** | 26,024 (8.35) | 37,442 (12.76) | 43,704 (16.04) | 32,448 (19.91) | 16,301 (24.07) | 9,362 (30.08) | <.0001 | |
| **Hypertension, n (%)** | 89,363 (28.66) | 113,742 (38.76) | 126,736 (46.52) | 88,649 (54.41) | 41,362 (61.09) | 21,720 (69.79) | <.0001 | |
| **Dyslipidemia, n (%)** | 56,454 (18.1) | 70,233 (23.93) | 76,057 (27.92) | 50,959 (31.28) | 23,412 (34.58) | 12,077 (38.8) | <.0001 | |
| **Smoking, n (%)** |  |  |  |  |  |  | <.0001 | |
| Non | 201,364 (64.58) | 172,312 (58.72) | 157,373 (57.77) | 91,191 (55.97) | 39,444 (58.25) | 19,153 (61.54) |  | |
| Ex | 42,497 (13.63) | 56,374 (19.21) | 57,709 (21.18) | 37,269 (22.87) | 14,931 (22.05) | 6,113 (19.64) |  | |
| Current | 67,965 (21.8) | 64,764 (22.07) | 57,331 (21.05) | 34,478 (21.16) | 13,337 (19.7) | 5,858 (18.82) |  | |
| **Alcohol, n (%)** |  |  |  |  |  |  | <.0001 | |
| Non | 200,374 (64.26) | 169,799 (57.86) | 155,509 (57.09) | 90,761 (55.7) | 39,285 (58.02) | 19,126 (61.45) |  | |
| Mild | 93,075 (29.85) | 101,695 (34.65) | 95,000 (34.87) | 57,048 (35.01) | 21,955 (32.42) | 8,938 (28.72) |  | |
| Heavy | 18,377 (5.89) | 21,956 (7.48) | 21,904 (8.04) | 15,129 (9.29) | 6,472 (9.56) | 3,060 (9.83) |  | |
| **Regular exercise, n (%)** | 67,332 (21.59) | 68,042 (23.19) | 62,426 (22.92) | 36,156 (22.19) | 13,956 (20.61) | 5,759 (18.5) | <.0001 | |
| **Cancer, n (%)** | 8,075 (2.59) | 6,116 (2.08) | 5,400 (1.98) | 3,176 (1.95) | 1,348 (1.99) | 596 (1.91) | <.0001 | |
| **ESRD, n (%)** | 340 (0.11) | 284 (0.1) | 234 (0.09) | 143 (0.09) | 66 (0.1) | 40 (0.13) | <.0001 | |
| **COPD, n (%)** | 20,888 (6.7) | 19,460 (6.63) | 18,985 (6.97) | 12,127 (7.44) | 5,469 (8.08) | 2,753 (8.85) | <.0001 | |
| **Stroke, n (%)** | 5,964 (1.91) | 6,880 (2.34) | 7,766 (2.85) | 5,371 (3.3) | 2,621 (3.87) | 1,429 (4.59) | <.0001 | |
| **LC, n (%)** | 1,331 (0.43) | 1,342 (0.46) | 1,316 (0.48) | 804 (0.49) | 344 (0.51) | 200 (0.64) | 0.1916 | |
| **HF, n (%)** | 2,000 (0.64) | 2,112 (0.72) | 2,234 (0.82) | 1,658 (1.02) | 875 (1.29) | 568 (1.82) | <.0001 | |
| **Dementia, n (%)** | 2,206 (0.71) | 1,854 (0.63) | 1,945 (0.71) | 1,289 (0.79) | 605 (0.89) | 355 (1.14) | <.0001 | |
| **CKD, n (%)** | 22,554 (7.23) | 24,946 (8.5) | 25,601 (9.4) | 16,616 (10.2) | 7,857 (11.6) | 4,124 (13.25) | <.0001 | |
| **Height, cm** | 159.69 ± 7.88 | 161.64 ± 8.08 | 162.42 ± 8.42 | 163.35 ± 8.68 | 163.34 ± 9.06 | 162.96 ± 9.41 | <.0001 | |
| **Weight, kg** | 54.1 ± 6.91 | 60.86 ± 7.16 | 65.14 ± 7.83 | 69.62 ± 8.51 | 73.48 ± 9.3 | 79.04 ± 10.9 | <.0001 | |
| **BMI, kg/m^2^** | 21.18 ± 2.01 | 23.26 ± 1.81 | 24.65 ± 1.88 | 26.04 ± 1.99 | 27.48 ± 2.15 | 29.71 ± 2.85 | <.0001 | |
| **WC, cm** | 72.33 ± 4.4 | 79.99 ± 2.86 | 84.9 ± 2.83 | 89.89 ± 2.74 | 94.61 ± 2.8 | 101.11 ± 4.42 | <.0001 | |
| **Glucose, mg/dL** | 97.5 ± 24.14 | 101.23 ± 26.83 | 103.64 ± 28.09 | 106.26 ± 30.08 | 108.89 ± 32.4 | 112.48 ± 35.05 | <.0001 | |
| **SBP, mmHg** | 122.1 ± 15.69 | 125.49 ± 15.41 | 127.43 ± 15.31 | 129.21 ± 15.31 | 130.6 ± 15.53 | 132.78 ± 15.89 | <.0001 | |
| **DBP, mmHg** | 75.71 ± 10.12 | 77.65 ± 10.02 | 78.74 ± 10 | 79.75 ± 10.1 | 80.46 ± 10.19 | 81.61 ± 10.44 | <.0001 | |
| **Total cholesterol, mg/dL** | 196.79 ± 36.6 | 200.33 ± 37.54 | 201.88 ± 38.18 | 202.26 ± 38.78 | 202.96 ± 39.67 | 203 ± 40.57 | <.0001 | |
| **HDL-C, mg/dL** | 59.35 ± 31.32 | 55.36 ± 29.94 | 53.44 ± 28.77 | 52.14 ± 28.03 | 51.64 ± 27.37 | 51.27 ± 26.8 | <.0001 | |
| **LDL-C, mg/dL** | 116.48 ± 37.68 | 118.91 ± 38.67 | 119.24 ± 39.22 | 118.65 ± 40.64 | 118.47 ± 40.92 | 117.85 ± 42.92 | <.0001 | |
| **Triglyceride^a^, mg/dL** | 98.13 (97.95 - 98.3) | 118.86 (118.63 - 119.09) | 132.11 (131.84 - 132.37) | 142.27 (141.9 - 142.63) | 148.53 (147.94 - 149.12) | 153.67 (152.78 - 154.56) | <.0001 | |
| **e-GFR, mL/min/1.73 m^2^** | 84.8 ± 32.47 | 83.28 ± 34.07 | 82.56 ± 35.51 | 81.88 ± 34.33 | 81.46 ± 37.47 | 80.94 ± 34.53 | <.0001 | |

Continuous variables were presented using mean and standard deviation. Categorical variables are expressed in numbers and percentages

^a^Geometric mean values for triglyceride

Abbreviations: DM, diabetes mellitus; ESRD, end-stage renal disease; COPD, chronic obstructive pulmonary disease; LC, liver cirrhosis; HF, hear failure; CKD, chronic kidney disease; BMI, body mass index; WC, waist circumference; SBP, systolic blood pressure; DBP, diastolic blood pressure; HDL, high-density lipoprotein cholesterol; LDL-C, low-density lipoprotein cholesterol; e-GFR, estimated glomerular filtration rate

**S4 Table.** **Incidence rate and hazard ratio for the risk of knee OA of the general obesity and/or central obesity composition according to age and sex composition**

|  |  |  | |  | |  |  | |  | |  | |  |  | |
| --- | --- | --- | --- | --- | --- | --- | --- | --- | --- | --- | --- | --- | --- | --- | --- |
|  | **General obesity** | | **Central obesity** | | **Total,  n** | | | **Knee OA,  n** | | **IR  (per 1,000)** | | **HR (95% CI)** | | |  |
|  |  |  |  |  |  |  |  |  |  |  |  | **Fully adjusted model** | | |  |

**Male**

| **50 - 59** | | | |  |  | |  | |  | | | ***P for interaction <0.0001*** | | | | | |  |  |
| --- | --- | --- | --- | --- | --- | --- | --- | --- | --- | --- | --- | --- | --- | --- | --- | --- | --- | --- | --- |
|  | No | No | 217,576 | | | 44,964 | | 28.049 | | 1 (ref.) | | | | | | |  |  |  |
|  |  | Yes | 13,659 | | | 3,264 | | 33.133 | | 1.157 (1.117, 1.199) | | | | | | |  |  |  |
|  | Yes | No | 68,762 | | | 16,848 | | 33.77 | | 1.2 (1.179, 1.222) | | | | | | |  |  |  |
|  |  | Yes | 75,644 | | | 20,739 | | 38.639 | | 1.354 (1.332, 1.377) | | | | | | |  |  |  |
| **60 - 69** | | | |  |  | | | | |  |  | |  | |  | | | |  |
|  | No | No | 122,243 | | | 36,704 | | 44.905 | | 1 (ref.) | | | | | | |  |  |  |
|  |  | Yes | 11,613 | | | 3,916 | | 52.169 | | 1.144 (1.106, 1.182) | | | | | | |  |  |  |
|  | Yes | No | 27,725 | | | 9,800 | | 53.68 | | 1.203 (1.176, 1.23) | | | | | | |  |  |  |
|  |  | Yes | 41,753 | | | 15,734 | | 59.119 | | 1.309 (1.284, 1.333) | | | | | | |  |  |  |
| **70 - 79** | | | |  |  | | | | |  |  | | |  | |  | | | |
|  | No | No | 49,300 | | | 15,613 | | 53.661 | | 1 (ref.) | | | | | | |  |  |  |
|  |  | Yes | 6,011 | | | 2,063 | | 60.481 | | 1.12 (1.07, 1.173) | | | | | | |  |  |  |
|  | Yes | No | 6,516 | | | 2,400 | | 61.834 | | 1.17 (1.12, 1.221) | | | | | | |  |  |  |
|  |  | Yes | 13,136 | | | 5,011 | | 65.805 | | 1.234 (1.195, 1.274) | | | | | | |  |  |  |
| **80 -** | | | |  |  | | | | |  |  | | |  | |  | | | |
|  | No | No | ,6780 | | | 1,704 | | 53.635 | | 1 (ref.) | | | | | | |  |  |  |
|  |  | Yes | 881 | | | 235 | | 56.244 | | 1.045 (0.911, 1.197) | | | | | | |  |  |  |
|  | Yes | No | 461 | | | 141 | | 61.863 | | 1.172 (0.987, 1.392) | | | | | | |  |  |  |
|  |  | Yes | 1,090 | | | 345 | | 64.412 | | 1.201 (1.07, 1.348) | | | | | | |  |  |  |

**Female**

| **50 - 59** | | | |  |  | |  | |  | | | ***P for interaction <0.0001*** | | | | | |  |  |
| --- | --- | --- | --- | --- | --- | --- | --- | --- | --- | --- | --- | --- | --- | --- | --- | --- | --- | --- | --- |
|  | No | No | 200,844 | | | 77,345 | | 58.159 | | 1 (ref.) | | | | | | |  |  |  |
|  |  | Yes | 8,929 | | | 4,097 | | 73.587 | | 1.231 (1.193, 1.27) | | | | | | |  |  |  |
|  | Yes | No | 46,698 | | | 22,927 | | 80.686 | | 1.376 (1.356, 1.396) | | | | | | |  |  |  |
|  |  | Yes | 40,038 | | | 21,886 | | 94.946 | | 1.595 (1.571, 1.619) | | | | | | |  |  |  |
| **60 - 69** | | | |  |  | | | | |  |  | |  | |  | | | |  |
|  | No | No | 76,443 | | | 38,882 | | 86.598 | | 1 (ref.) | | | | | | |  |  |  |
|  |  | Yes | 7,789 | | | 4,477 | | 105.139 | | 1.193 (1.156, 1.23) | | | | | | |  |  |  |
|  | Yes | No | 18,770 | | | 11,427 | | 114.622 | | 1.323 (1.296, 1.351) | | | | | | |  |  |  |
|  |  | Yes | 25,794 | | | 16,472 | | 126.422 | | 1.448 (1.422, 1.475) | | | | | | |  |  |  |
| **70 - 79** | | | |  |  | | | | |  |  | | |  | |  | | | |
|  | No | No | 25,956 | | | 13,089 | | 93.624 | | 1 (ref.) | | | | | | |  |  |  |
|  |  | Yes | 4,223 | | | 2,277 | | 105.175 | | 1.109 (1.06, 1.159) | | | | | | |  |  |  |
|  | Yes | No | 4,345 | | | 2,618 | | 121.227 | | 1.306 (1.253, 1.362) | | | | | | |  |  |  |
|  |  | Yes | 9,208 | | | 5,590 | | 125.036 | | 1.336 (1.295, 1.379) | | | | | | |  |  |  |
| **80 -** | | | |  |  | | | | |  |  | | |  | |  | | | |
|  | No | No | 4,879 | | | 1,531 | | 66.229 | | 1 (ref.) | | | | | | |  |  |  |
|  |  | Yes | 966 | | | 354 | | 77.281 | | 1.168 (1.041, 1.311) | | | | | | |  |  |  |
|  | Yes | No | 391 | | | 155 | | 79.555 | | 1.202 (1.019, 1.418) | | | | | | |  |  |  |
|  |  | Yes | 1,040 | | | 442 | | 88.547 | | 1.351 (1.215, 1.502) | | | | | | |  |  |  |

Note: Fully adjusted model was adjusted for age, sex, income, smoking, alcohol intake, regular exercises, diabetes, hypertension, dyslipidemia, cancer, end-stage renal disease, chronic obstructive pulmonary disease, stroke, liver cirrhosis, hear failure, dementia and chronic kidney disease.

Abbreviations: HR, Hazard ratio; CI, confidence interval; OA, osteoarthritis; IR, incidence rate.

**S5 Table.** **Baseline characteristics of study participants according to the change in general obesity status**

|  | **Change in general obesity (Pre / Post)** | | | | |
| --- | --- | --- | --- | --- | --- |
|  | **No / No** | **No / Yes** | **Yes / No** | **Yes / Yes** | P-value |
|  | (n=430197) | (n=37104) | (n=38477) | (n=190452) |  |
| **Age** |  |  |  |  |  |
| mean | 58.47 ± 7.43 | 58.02 ± 6.97 | 58.62 ± 7.25 | 57.84 ± 6.69 | <.0001 |
| Categories, n (%) |  |  |  |  |  |
| 50 - 59 | 262,360 (60.99) | 23,465 (63.24) | 22,854 (59.4) | 121,124 (63.6) | <.0001 |
| 60 - 69 | 122,119 (28.39) | 10,519 (28.35) | 11,665 (30.32) | 55,341 (29.06) |  |
| 70 - 79 | 41,289 (9.6) | 2,868 (7.73) | 3,655 (9.5) | 13,199 (6.93) |  |
| 80 - | 4,429 (1.03) | 252 (0.68) | 303 (0.79) | 788 (0.41) |  |
| **Male, n (%)** | 249,295 (57.95) | 23,195 (62.51) | 24,326 (63.22) | 126,053 (66.19) | <.0001 |
| **Low income** <**25%, n (%)** | 99,390 (23.1) | 8,587 (23.14) | 8,853 (23.01) | 42,873 (22.51) | <.0001 |
| **Type 2 DM, n (%)** | 49,431 (11.49) | 4,844 (13.06) | 7,289 (18.94) | 33,954 (17.83) | <.0001 |
| **Hypertension, n (%)** | 147,039 (34.18) | 15,983 (43.08) | 18,636 (48.43) | 101,814 (53.46) | <.0001 |
| **Dyslipidemia, n (%)** | 93,003 (21.62) | 9,873 (26.61) | 11,236 (29.2) | 59,369 (31.17) | <.0001 |
| **Smoking, n (%)** |  |  |  |  | <.0001 |
| Non | 257,785 (59.92) | 20,891 (56.3) | 22,416 (58.26) | 106,017 (55.67) |  |
| Ex | 78,769 (18.31) | 7,785 (20.98) | 8,756 (22.76) | 46,753 (24.55) |  |
| Current | 93,643 (21.77) | 8,428 (22.71) | 7,305 (18.99) | 37,682 (19.79) |  |
| **Alcohol, n (%)** |  |  |  |  | <.0001 |
| Non | 254,095 (59.06) | 20,774 (55.99) | 21,842 (56.77) | 102,372 (53.75) |  |
| Mild | 147,104 (34.19) | 13,404 (36.13) | 13,621 (35.4) | 70,745 (37.15) |  |
| Heavy | 28,998 (6.74) | 2,926 (7.89) | 3,014 (7.83) | 17,335 (9.1) |  |
| **Regular exercise, n (%)** | 97,176 (22.59) | 8,712 (23.48) | 8,964 (23.3) | 45,856 (24.08) | <.0001 |
| **Cancer, n (%)** | 9,330 (2.17) | 680 (1.83) | 702 (1.82) | 3,029 (1.59) | <.0001 |
| **ESRD, n (%)** | 334 (0.08) | 26 (0.07) | 24 (0.06) | 106 (0.06) | 0.0239 |
| **COPD, n (%)** | 28,312 (6.58) | 2,517 (6.78) | 2,570 (6.68) | 12,078 (6.34) | 0.0004 |
| **Stroke, n (%)** | 9,060 (2.11) | 965 (2.6) | 1,037 (2.7) | 5,135 (2.7) | <.0001 |
| **LC, n (%)** | 1,755 (0.41) | 173 (0.47) | 164 (0.43) | 765 (0.4) | 0.3225 |
| **HF, n (%)** | 2,623 (0.61) | 243 (0.65) | 308 (0.8) | 1,623 (0.85) | <.0001 |
| **Dementia, n (%)** | 2,269 (0.53) | 202 (0.54) | 228 (0.59) | 874 (0.46) | 0.0004 |
| **CKD, n (%)** | 34,304 (7.97) | 3,165 (8.53) | 3,619 (9.41) | 17,889 (9.39) | <.0001 |
| **Height, cm** | 161.89 ± 8.16 | 162.57 ± 8.26 | 162.07 ± 8.51 | 162.92 ± 8.4 | <.0001 |
| **Weight, kg** | 58.23 ± 7.66 | 64.03 ± 6.89 | 67.68 ± 7.22 | 72.31 ± 8.56 | <.0001 |
| **2009 Health examination** |  |  |  |  |  |
| **BMI, kg/m^2^** | 22.15 ± 1.78 | 24.16 ± 0.82 | 25.71 ± 1.04 | 27.18 ± 1.81 | <.0001 |
| **WC, cm** | 78.21 ± 6.7 | 83.22 ± 5.74 | 85.66 ± 5.77 | 89.09 ± 6.58 | <.0001 |
| **2011 Health examination** |  |  |  |  |  |
| **BMI, kg/m^2^** | 22.12 ± 1.8 | 25.68 ± 1 | 24.1 ± 0.87 | 27.2 ± 1.83 | <.0001 |
| **WC, cm** | 78.18 ± 6.71 | 85.63 ± 5.81 | 83.13 ± 5.75 | 89.24 ± 6.59 | <.0001 |
| **Glucose, mg/dL** | 99.81 ± 25.14 | 101.96 ± 28.75 | 105.25 ± 28.33 | 104.99 ± 27.63 | <.0001 |
| **SBP, mmHg** | 123.89 ± 15.35 | 125.88 ± 14.95 | 128.21 ± 15.4 | 129 ± 14.92 | <.0001 |
| **DBP, mmHg** | 76.74 ± 9.99 | 78.17 ± 9.87 | 79.3 ± 10.06 | 80.06 ± 9.93 | <.0001 |
| **Total cholesterol, mg/dL** | 198.91 ± 36.8 | 201.02 ± 38.14 | 202.76 ± 38.04 | 202.71 ± 38.14 | <.0001 |
| **HDL-C, mg/dL** | 56.75 ± 29.73 | 54.67 ± 30.9 | 52.77 ± 27.85 | 52.1 ± 27.37 | <.0001 |
| **LDL-C, mg/dL** | 117.89 ± 38.08 | 119.17 ± 38.93 | 119.51 ± 39.12 | 119.25 ± 39.79 | <.0001 |
| **Triglyceride^a^, mg/dL** | 110.69 (110.51 - 110.87) | 123.58 (122.91 - 124.27) | 136.87 (136.14 - 137.61) | 141.5 (141.16 - 141.84) | <.0001 |
| **e-GFR, mL/min/1.73 m^2^** | 83.87 ± 35.09 | 82.8 ± 34.44 | 82.1 ± 33.3 | 81.75 ± 35.14 | <.0001 |

Continuous variables were presented using mean and standard deviation. Categorical variables are expressed in numbers and percentages

(Pre / Post) means obesity status in the preceding and subsequent health examination.

^a^Geometric mean values for triglyceride

Abbreviations: DM, diabetes mellitus; ESRD, end-stage renal disease; COPD, chronic obstructive pulmonary disease; LC, liver cirrhosis; HF, hear failure; CKD, chronic kidney disease; BMI, body mass index; WC, waist circumference; SBP, systolic blood pressure; DBP, diastolic blood pressure; HDL, high-density lipoprotein cholesterol; LDL-C, low-density lipoprotein cholesterol; e-GFR, estimated glomerular filtration rate

**S6 Table.** **Baseline characteristics of study participants according to the change in central obesity status**

|  | **Change in central obesity (Pre / Post)** | | | | |
| --- | --- | --- | --- | --- | --- |
|  | **No / No** | **No / Yes** | **Yes / No** | **Yes / Yes** | P-value |
|  | (n=488909) | (n=55497) | (n=54363) | (n=97461) |  |
| **Age** |  |  |  |  |  |
| mean | 57.91 ± 7.13 | 58.81 ± 7.24 | 59.15 ± 7.36 | 59.31 ± 7.32 | <.0001 |
| Categories, n (%) |  |  |  |  |  |
| 50 - 59 | 313,135 (64.05) | 32,287 (58.18) | 30,694 (56.46) | 53,687 (55.09) | <.0001 |
| 60 - 69 | 132,221 (27.04) | 17,477 (31.49) | 17,507 (32.2) | 32,439 (33.28) |  |
| 70 - 79 | 39,653 (8.11) | 5,258 (9.47) | 5,624 (10.35) | 10,476 (10.75) |  |
| 80 - | 3,900 (0.8) | 475 (0.86) | 538 (0.99) | 859 (0.88) |  |
| **Male, n (%)** | 287,456 (58.8) | 33,901 (61.09) | 34,390 (63.26) | 67,122 (68.87) | <.0001 |
| **Low income** <**25%, n (%)** | 112,025 (22.91) | 12,633 (22.76) | 12,691 (23.34) | 22,354 (22.94) | 0.1014 |
| **Type 2 DM, n (%)** | 54,487 (11.14) | 8,534 (15.38) | 10,372 (19.08) | 22,125 (22.7) | <.0001 |
| **Hypertension, n (%)** | 170,911 (34.96) | 26,616 (47.96) | 27,877 (51.28) | 58,068 (59.58) | <.0001 |
| **Dyslipidemia, n (%)** | 108,299 (22.15) | 15,955 (28.75) | 16,477 (30.31) | 32,750 (33.6) | <.0001 |
| **Smoking, n (%)** |  |  |  |  | <.0001 |
| Non | 292,248 (59.78) | 32,055 (57.76) | 31,248 (57.48) | 51,558 (52.9) |  |
| Ex | 93,226 (19.07) | 11,602 (20.91) | 12,540 (23.07) | 24,695 (25.34) |  |
| Current | 103,435 (21.16) | 11,840 (21.33) | 10,575 (19.45) | 21,208 (21.76) |  |
| **Alcohol, n (%)** |  |  |  |  | <.0001 |
| Non | 285,313 (58.36) | 31,613 (56.96) | 30,666 (56.41) | 51,491 (52.83) |  |
| Mild | 170,615 (34.9) | 19,329 (34.83) | 19,208 (35.33) | 35,722 (36.65) |  |
| Heavy | 32,981 (6.75) | 4,555 (8.21) | 4,489 (8.26) | 10,248 (10.51) |  |
| **Regular exercise, n (%)** | 114,191 (23.36) | 12,789 (23.04) | 12,102 (22.26) | 21,626 (22.19) | <.0001 |
| **Cancer, n (%)** | 10,077 (2.06) | 1,015 (1.83) | 961 (1.77) | 1,688 (1.73) | <.0001 |
| **ESRD, n (%)** | 344 (0.07) | 43 (0.08) | 27 (0.05) | 76 (0.08) | 0.211 |
| **COPD, n (%)** | 30,473 (6.23) | 3,855 (6.95) | 3,860 (7.1) | 7,289 (7.48) | <.0001 |
| **Stroke, n (%)** | 9,740 (1.99) | 1,549 (2.79) | 1,517 (2.79) | 3,391 (3.48) | <.0001 |
| **LC, n (%)** | 1,946 (0.4) | 239 (0.43) | 237 (0.44) | 435 (0.45) | 0.0964 |
| **HF, n (%)** | 2,825 (0.58) | 446 (0.8) | 480 (0.88) | 1,046 (1.07) | <.0001 |
| **Dementia, n (%)** | 2,239 (0.46) | 344 (0.62) | 355 (0.65) | 635 (0.65) | <.0001 |
| **CKD, n (%)** | 38,576 (7.89) | 5,052 (9.1) | 5,190 (9.55) | 10159 (10.42) | <.0001 |
| **Height, cm** | 161.55 ± 8.02 | 162.9 ± 8.48 | 163.11 ± 8.56 | 164.71 ± 8.58 | <.0001 |
| **Weight, kg** | 59.51 ± 8.21 | 66.93 ± 8.15 | 68.37 ± 8.4 | 74.64 ± 9.11 | <.0001 |
| **2009 Health examination** |  |  |  |  |  |
| **BMI, kg/m^2^** | 22.75 ± 2.23 | 25.17 ± 1.96 | 25.64 ± 1.96 | 27.48 ± 2.36 | <.0001 |
| **WC, cm** | 78.38 ± 6.2 | 83.56 ± 4.35 | 90.54 ± 3.45 | 93.56 ± 4.93 | <.0001 |
| **2011 Health examination** |  |  |  |  |  |
| **BMI, kg/m^2^** | 22.72 ± 2.25 | 25.68 ± 1.97 | 25.08 ± 1.97 | 27.5 ± 2.39 | <.0001 |
| **WC, cm** | 78.35 ± 6.22 | 90.49 ± 3.51 | 83.6 ± 4.44 | 93.67 ± 4.97 | <.0001 |
| **Glucose, mg/dL** | 99.78 ± 24.57 | 103.18 ± 27.96 | 105.58 ± 29.06 | 107.95 ± 30.73 | <.0001 |
| **SBP, mmHg** | 124.22 ± 15.28 | 127.33 ± 15.08 | 128.64 ± 15.14 | 130.09 ± 15.08 | <.0001 |
| **DBP, mmHg** | 77.06 ± 9.99 | 78.78 ± 9.91 | 79.51 ± 10.06 | 80.46 ± 10.03 | <.0001 |
| **Total cholesterol, mg/dL** | 199.47 ± 36.8 | 201.85 ± 38.1 | 203.05 ± 38.63 | 201.89 ± 38.76 | <.0001 |
| **HDL-C, mg/dL** | 56.41 ± 29.58 | 53.48 ± 29.81 | 52.44 ± 27.71 | 51.27 ± 26.76 | <.0001 |
| **LDL-C, mg/dL** | 118.34 ± 38.01 | 119.34 ± 39.38 | 119.42 ± 39.58 | 117.72 ± 40.88 | <.0001 |
| **Triglyceride^a^, mg/dL** | 112.39 (112.22 - 112.56) | 132.43 (131.84 - 133.01) | 139.98 (139.35 - 140.61) | 148.85 (148.36 - 149.34) | <.0001 |
| **e-GFR, mL/min/1.73 m^2^** | 83.54 ± 34.3 | 82.9 ± 35.8 | 82.51 ± 39.71 | 81.61 ± 35.06 | <.0001 |

Continuous variables were presented using mean and standard deviation. Categorical variables are expressed in numbers and percentages

(Pre / Post) means obesity status in the preceding and subsequent health examination.

^a^Geometric mean values for triglyceride

Abbreviations: DM, diabetes mellitus; ESRD, end-stage renal disease; COPD, chronic obstructive pulmonary disease; LC, liver cirrhosis; HF, hear failure; CKD, chronic kidney disease; BMI, body mass index; WC, waist circumference; SBP, systolic blood pressure; DBP, diastolic blood pressure; HDL, high-density lipoprotein cholesterol; LDL-C, low-density lipoprotein cholesterol; e-GFR, estimated glomerular filtration rate
